# Alveolar Abscess

**Published:** 1892-05

**Authors:** Edward C. Kirk


### ALVEOLAR ABSCESS.[1]

BY EDWARD C. KIRK, D.D.S.

Mr. President and Gentlemen,—I am compelled to preface my remarks with an apology for not presenting to you a formally-prepared essay, as I have been unable from lack of time to go through the mechanical work of writing a paper; so I will ask your indulgence while I speak from my rough notes.

It would be entirely superfluous to bring before this audience a description of alveolar abscess, as we meet it ordinarily. The clinical expressions of alveolar abscess are well known to all of us, but there are some forms of this disorder that occur infrequently, which have a special importance because of their possible fatal termination; and as the methods of treatment which we should adopt in cases of this sort are so entirely different from those which we ordinarily pursue, it seems not only worth while, but necessary, that we should be able to recognize them from their clinical expression in the very first stages.

Alveolar abscess, you know, starting as an acute attack of local inflammation, runs through a period of from twenty-four hours to a week, which may be required to bring it through the stage of pus-formation. I refer now to the class of cases where we have a large collection of pus, which is of a creamy, yellow character, which, when this is once evacuated, so far as the acute stage is concerned, rapidly subsides. That is what I recognize as the ordinary form.

About two years ago I was called to see a patient, at his house, suffering from alveolar abscess under rather peculiar circumstances. He had been for eighteen months running down in health; his special disorder was a profound nervous prostration, or neurasthenia. The nervous trouble had been progressing until his life was despaired of, but he had been slowly rallying from this. His disorder ran through a period of nearly two years from its inception to termination. It was during this time that I was called in to relieve him of pain in one of his molar teeth. I found a lower sixth-year molar on the left side presenting the symptoms of alveolar abscess as we ordinarily understand it. The seat of inflammation was about the anterior root. He was confined to his bed, greatly prostrated, and, of course, my efforts at treatment were necessarily limited by the man's broken-down condition and my

[1] Read before the Odontological Society of Pennsylvania, March 12, 1892.

inability to properly get at the seat of the difficulty with remedies, owing to the lack of proper appliances for the treatment of such a case by the usual methods in the office; however, the inflammation ran its usual course, and in three or four days the sack was evacuated by lancing, he was relieved, and rapidly recovered from the effect of the attack. The cavity was on the mesial approximal surface, and there was ready access to the pulp-chamber. All I was able to do at the time was to sterilize the nerve-canal and pulp-chamber with tincture of iodine; the tooth was stopped afterwards with gutta-percha, after the canals had been filled with a paste made of hydronaphthol and eugenol.

The long period of his illness made it some months before I saw the patient again, though he came to me as soon as he was able to be out. I examined his teeth and found some slight carious places that needed attention, but overlooked this sixth-year molar, as it was absolutely comfortable, rendering him good service, and had all the appearances of a perfectly healthy, pulpless tooth. The only peculiarity was that the tooth was slightly raised at its mesial aspect, probably from the deposition of plastic material about the apex of the anterior root of the one which had been affected; otherwise the tooth was, to all appearances, healthy. There was no inflammation of the adjacent soft tissues, and no pain. His health became much better, he was able to attend to his business, and was seemingly on the high road to health. Last July he spent the Fourth at Atlantic City, and had "taken cold," as he expressed it. He returned on the 5th or 6th, and came to me complaining of pain in the region of the same tooth that I had treated the year before. I immediately inspected the tooth and found some portions of the paste remaining, but apparently disintegrating, and it was altogether in an unsatisfactory condition. On the border of the inferior maxilla, on a vertical line immediately below the tooth, was a small inflammatory bunch, which was quite dense and hard and attached to the bone, apparently, and immovable. The patient was suffering considerable pain at the time, though an examination showed no inflammation of the soft tissues surrounding the tooth. It was but slightly sore on pressure. The sixth-year molar above had been lost, and considering the critical condition of the man's nervous system, together with the location of the inflammation, which was considerably outside the region of the mouth, I reasoned that if pus had to burrow its way back into the mouth, it would take a long time, and that he was not in a fit condition to undergc the acute suffering that would be entailed. I therefore advised him to have the tooth removed, which was done by Dr. J. D. Thomas, under nitrous oxide. The next morning, after the removal of the tooth, the patient presented himself for inspection. I found the inflamed area somewhat enlarged, but only slightly, and I attributed it to the irritation caused by the extraction of the tooth. The socket contained two pledgets of cotton, which had been saturated with phénol sodique.

I inquired whether there had been any flow of pus following the extraction of the tooth. He said there had not, nor had Dr. Thomas observed any. I assured him of my belief that the extraction of the tooth would remove the difficulty, and he would soon feel better. The socket was washed out, and cotton with phénol sodique was reapplied. The next day I was sent for by the patient, and found him at his house confined to bed, having had a very painful and restless night suffering from a chill, and again presenting symptoms of profound nervous depression. The inflamed area had enlarged considerably, the skin was tense and glistening over the lump. Examination of the mouth revealed entire absence of inflammation of the soft tissues within the oral cavity. Taking into consideration his general condition, the symptoms indicating where the abscess was tending to form, it became very clear to my mind that to try to establish an exit of pus within the mouth would be dangerous or fatal in his case, because of his lack of vitality and the length of time that would necessarily be required to evacuate the pus from within the mouth, so I decided to poultice the swelling and establish the exit of pus from the outside. He was given tonic treatment, stimulants and quinine, and some morphia to relieve the pain. I then prescribed flaxseed poultices for twenty-four hours, at the expiration of which I found the swollen territory considerably enlarged, involving by this time the whole cheek and down the neck, nearly to the border of the clavicle; the skin had become copper-colored, and the whole surface was hard and tense and the patient very much exhausted from pain. No part of the surface seemed soft,—no place where a most careful examination showed pus to be determining. There seemed to be nothing to do but continue the application of poultices, which was done until the next morning, when by a careful examination with the index finger of the left hand in the mouth, and by careful palpation with the fingers of the right hand over the inflamed area in every direction, I found one small spot about a half-inch in diameter, where there was a slight pulsation observable. Into this I passed a lancet and carried its point to the free border of the inferior maxilla, immediately below the position of the roots of the affected molar. This was followed by a very slight exudatian of pus, just enough to bring out on the knife-blade. The exudation was of a peculiar character,—glutinous, sticky, and albuminous in appearance, streaked with blood, and only a very slight amount of pus as we usually see it, that which we know as the laudable pus ordinarily evacuated from alveolar abscesses. Poulticing was continued constantly, and the constitutional treatment kept up. The discharge continued of the same character,—slimy and glutinous. The cavity made by the incision was washed with an antiseptic of phénol sodique in the proportion of a tablespoonful to two-thirds of a tumblerful of warm water. This gave some relief, but he was still suffering intensely from pain; the constitutional disturbance was very marked, and was out of all proportion to the pain he was suffering. The even character of the surface of the inflamed territory, which had characterized the condition up to date, now commenced to take on another form: there seemed to be centres where suppuration would present some three or four of them, giving it a hilly or boggy appearance, the surface appearing quite irregular. About four days later, after continued poulticing, a centre of suppuration formed about an inch and a half above and back of the first. This, in course of time, was opened and evacuated. Neither in washing it out nor by exploring with a probe was I able to detect any connection between the two openings. In the course of a week another centre of inflammation suppurated, which was still higher up, just below the ear. This was evacuated. By this time the two original pus-cavities became united, so that I could pass antiseptic solutions in at the second opening and out through the first. The jaws all this time were quite fixed, the patient being unable to open them more than an eighth or three-sixteenths of an inch; his throat was also very much swollen, and he had, so far as I could determine, all the physical signs of tonsillitis. The throat seemed to be closing up, so that it became almost impossible for him to swallow. I watched very carefully to see if there was any discharge of pus from the inside, but this did not occur, as far as I am aware. Later on, another centre of suppuration occurred upon the neck, about two inches below the border of the jaw. This was evacuated in the same way as the others. By this time the original opening commenced to heal, and there was a noticeable turn for the better. His general appearance was improved, and it was evident that the inflammation was subsiding. This state of affairs continuing, he was sent away to a summer resort, and during the summer his health returned to about normal. There was still some discharge at the first of September from the original opening, but the others had healed up. I saw him in October, and there was at that time a slight periodical discharge from the original fistula.

By this time he was able to open the mouth normally, and I made a careful examination. You will recollect that the sixth-year molar had been extracted; the second biscuspid was a pulpless tooth. I knew that to be in good order, as it was perfectly filled and gave no trouble. The twelfth-year molar up to the time of the first attack of alveolar abscess had been vital, and it presented all the appearances of a vital tooth at the time of my last examination in October. However, on the buccal side of this twelfth-year molar there was a fistulous opening, and extending from it was a red line along the gum to a point about opposite the position of the sixth-year molar which had been extracted. I suspected from this that pus had burrowed along under the gum-tissues, and had found exit at the fistulous opening alluded to alongside the twelfth-year molar. I did not suspect that the twelfth-year molar was devitalized, as it was without a cavity or any defect. Had my suspicions as to its vitality been raised, I should have attributed the fistulous opening there to an abscess from that tooth, but I reasoned that necrosis of a portion of the alveolar process had taken place, and the discharge therefrom was finding exit alongside the twelfth-year molar.

You will remember that at this time every symptom of inflammatory action had subsided, with the exception of a slight scar upon the lower part of the face, which had resulted from the first incision. Once in ten days it would open and then heal up again. I attributed this to the existence of a certain amount of necrosed bone. It seemed to me almost impossible to have had such an active inflammation in this connection without some necrosis of the bone taking place. I tested the twelfth-year molar, and it seemed to respond to all the tests of a vital tooth, but the patient was very nervous, and it was difficult to decide. However, the problem solved itself in a most unexpected way. The articulation of the upper twelfth-year molar to the lower was very close, the palatal cusp of the upper fitting into a depression in the grinding surface of the lower like a wedge, and during a meal he suddenly bit down on something hard and split this apparently healthy lower twelfth-year molar through its entire antero-posterior diameter, which resulted in my discovering that it was absolutely pulpless. It was therefore at once removed, and within a week afterwards the fistulous opening on the face healed, and all traces of discharge ceased. I have examined the case carefully since then, and am unable to detect any fistula in the mouth, and he is to all appearances quite well. Dr. Peirce saw the case with me in consultation, and approved the treatment which had been carried out; he believed in the existence of some necrosed bone. I have not been able to discover it so far, although Dr. Peirce believes he has detected a point where there is some slight necrosis in the alveolar border.

The case ran over a period of nearly four months before recovery was complete. I want especially to emphasize the infectious character of the inflammation in the case as I have presented it to you. It seems perfectly evident to me that the septic material, which was the primary irritant in this case, was, if produced by micro-organisms from an infective pathogenic germ, different from that which produces ordinary abscess, the staphylococcus pyogenes. I fully believe that, if the case had not been treated upon general surgical principles by free evacuation of the discharge, and by washing out the pus-cavities with antiseptic solutions, and the patient treated constitutionally with stimulants and tonics, he would have died.

I wish to again call attention to the character of the pus-discharge. It was at no time of a creamy yellow color, but clear, viscid, and albuminous in character. It seems to me that this clinical point may be related to the kind of micro-organisms producing it.

If you will examine the literature of the subject, you will find that the effect produced by the action of various micro-organisms upon a given pabulum widely differs and varies in its expression, along with the other vital phenomena, according to the kind of organism or the class to which it belongs. From this view it is of interest to note that in the history of the case two distinct kinds of inflammatory action, presenting very different clinical phenomena, occurred in the same patient; both of these might be regarded as alveolar abscesses; yet one was benign in character, the other attended with alarming and unusual symptoms. The infectious character of the last attack was plainly evident, and if due to the invasion of a specific pathogenic germ, as the researches of Miller warrant us in believing, the unusual character of the discharge, perhaps, may bear a significant relation to the diagnosis in such cases, as its nature would seem to depend upon the character of the invading micro-organism, rather than upon the systemic condition of the patient. This view, I think, is sustained by the occurrence of two distinct forms of suppurative inflammation in the same patient, and involving the same territory,—the discharged pus being in each instance widely different in character; that is, the pabulum being the same, and the invading organism different, resulted, among other things, in a dissimilarity in their waste products.

The case which I have imperfectly presented is, I think, important. It is of a kind which, fortunately, occurs rarely, and I have felt justified in bringing it before you, with a view to calling out some expression of opinion upon this class of cases.

